# Exploring Antimalarial Herbal Plants across Communities in Uganda Based on Electronic Data

**DOI:** 10.1155/2019/3057180

**Published:** 2019-09-15

**Authors:** Denis Okello, Youngmin Kang

**Affiliations:** ^1^Korean Convergence Medicine Major, University of Science and Technology (UST), Daejeon, Republic of Korea; ^2^Gombe Secondary School, P. O. Box 192, Butambala, Mpigi, Uganda; ^3^Herbal Medicine Research Division, Korea Institute of Oriental Medicine (KIOM), Yuseongdae-ro, Yuseong-gu, Daejeon 34054, Republic of Korea

## Abstract

Malaria is one of the most rampant diseases today not only in Uganda but also throughout Africa. Hence, it needs very close attention as it can be severe, causing many deaths, especially due to the rising prevalence of pathogenic resistance to current antimalarial drugs. The majority of the Ugandan population relies on traditional herbal medicines for various health issues. Thus, herein, we review various plant resources used to treat malaria across communities in Uganda so as to provide comprehensive and valuable ethnobotanical data about these plants. Approximately 182 plant species from 63 different plant families are used for malaria treatment across several communities in Uganda, of which 112 plant species have been investigated for antimalarial activities and 96% of the plant species showing positive results. Some plants showed very strong antimalarial activities and could be investigated further for the identification and validation of potentially therapeutic antimalarial compounds. There is no record of an investigation of antimalarial activity for approximately 39% of the plant species used for malaria treatment, yet these plants could be potential sources for potent antimalarial remedies. Thus, the review provides guidance for areas of further research on potential plant resources that could be sources of compounds with therapeutic properties for the treatment of malaria. Some of the plants were investigated for antimalarial activities, and their efficacy, toxicity, and safety aspects still need to be studied.

## 1. Introduction

**Figure 1:**
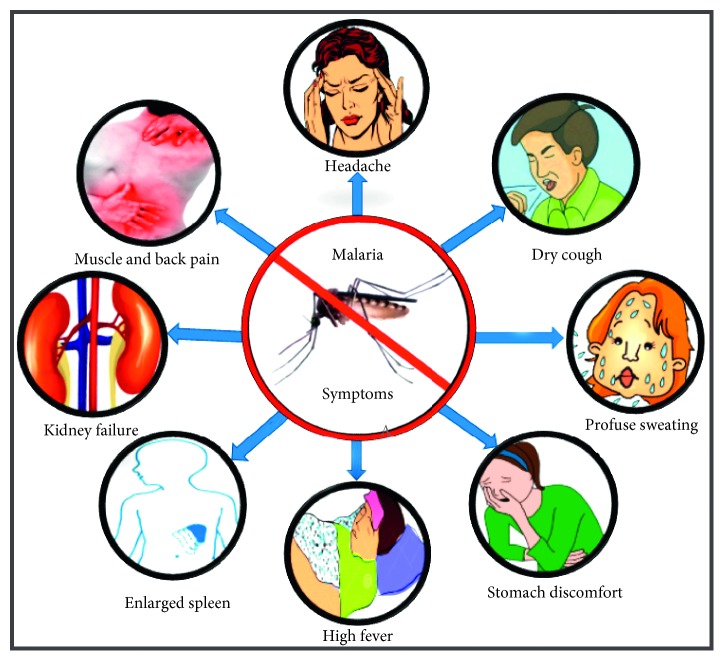
Illustration of some common symptoms of malaria.

Malaria, a dangerous and life-threatening disease caused by *Plasmodium* parasites is spread to humans through bites of infected female *Anopheles* mosquitoes [1]. It is one of the most widespread diseases today not only in Uganda but also throughout Africa. Hence, careful monitoring of malaria is required as the disease can be severe and can cause many deaths, especially due to the increasing prevalence of resistance to current antimalarial drugs. Among the five parasitic species that cause malaria to humans, *Plasmodium falciparum* and *Plasmodium vivax* are the deadliest [2, 3]. *P. falciparum* and *P. vivax* being the most prevalent malaria parasites in sub-Saharan Africa and regions of the Americas, respectively, were responsible for about 99.7% and 74.1% of malaria cases in 2017 [4]. In Southeast Asia, *Plasmodium knowlesi* is the most common cause of malaria, accounting for up to 70% of malaria cases, although it has been known to infect Old-World monkeys more [5]. Two other species of *Plasmodium*, *Plasmodium malariae* and *Plasmodium ovale*, generally cause mild fevers. Approximately 216 million malaria cases were registered in 2016, with a death toll of up to 445,000 [1]. According to the World Health Organization [6], the incidence of malaria in Uganda, at 47.8%, was the highest worldwide in 2005. According to Njoroge and Bussman [7], malaria is responsible for one to two million deaths annually in Africa. Typical symptoms of malaria include high fever, fatigue, headache, muscle ache, nausea, abdominal discomfort, and profuse sweating. However, in extreme cases and cases of prolonged illness without treatment, brain tissue injury, pulmonary edema, kidney failure, severe anemia, yellow discoloration of the skin, and low blood sugar may be noted (Figure 1) [1, 2]. In Uganda, malaria is one of the major causes of illness and death [7]. Statistically, it accounts for 46% of children's sicknesses, almost 40% of outpatient visits to hospitals and clinics, 25% of hospital admissions, 14% of inpatient deaths, and approximately 23% of infant mortalities [7].

In different parts of the world, the use of herbs and herbal extracts in the management and treatment of malaria is very common since herbs are cheap and readily available besides being effective. In fact, the use of herbal medicine for treatment worldwide is on the rise. Over 80% of the Ugandan population relies directly on herbal plants for their health care primarily [8]. A great majority of the population uses traditional herbal medicines because of their confirmed therapeutic value [8]. The increase in preference for herbal remedies coupled with resistance exhibited by pathogenic strains, including *Plasmodium* species, to the modern drugs available is the driving force behind researchers' interest in herbal plants for possible alternatives for more effective antimalarial drugs [9, 10].

This review was aimed at providing comprehensive ethnobotanical information about various plant resources with antimalarial properties that are primarily used to manage and treat malaria across communities in Uganda, based on which further evaluation of these plants such as those of their efficacy and safety for the treatment of malaria may be based.

## 2. Methods and Materials

In the review, the data search processes employed by Komakech et al. [11] were modified to gather information on herbal plants for malaria treatment in Uganda from peer-reviewed articles in English published in scientific journals and other verifiable databases, with a focus on plant species and families, plant parts used, antimalarial activities of the extracts from herbal plants, and mechanisms of action of novel antimalarial phytochemicals and derivatives. Electronic literature databases such as PubMed, Medline, Scopus, SciFinder, Google Scholar, and Science Direct were carefully searched for suitable information. The following words were used as key search terms: (“Herbal medicine in Uganda” OR “Herbs in Uganda” OR “Traditional remedies in Uganda” OR “Natural remedies in Uganda” OR “Anti-malarial herbs in Uganda” OR “Anti-malarial plants in Uganda” OR “Ugandan herbs” OR “Ugandan ethno-medicine” OR “Ugandan phyto-medicine”), AND (“anti-plasmodial activities” OR “anti-malarial activities” OR “anti-plasmodial effects” OR “anti-malarial effects” OR “malaria treatment” OR “malaria management”) OR (“Malaria in Uganda” AND “prevalence” OR “occurrence” OR “distribution” OR “herbal treatment” OR “herbal remedies” OR “phyto-medicine” OR “phyto remedy” OR “plant parts used for treatment”) OR (Phytochemicals for malaria treatment OR Artemisinins OR Quinine OR Noble anti-malarial compounds OR Plant derived anti-malarial compounds AND mechanisms of action OR modes of action) OR (“Malaria herbal medicine in Uganda” OR “Herbal medicine in Uganda” OR “Herbal malaria remedy in Uganda” OR “Natural malaria medicine in Uganda” OR “Traditional malaria herbal medicine” OR “Malaria herbal recipe” AND “dosage” OR “dose” OR “dose given” OR “mode of administration” OR “means of traditional extraction” OR “traditional extraction” OR “Toxicity” OR “Safety and toxicity” OR “Policy framework” OR “other ethno-pharmacological uses” OR “other ethno-pharmacological utilizations” OR “other ethno-medicinal uses”). The information gathered was verified separately for its reliability; any discrepancies discovered were resolved by discussions between the authors. Thereafter, these data were summarized and analyzed, and comparisons were made to draw conclusions.

## 3. Prevalence of Malaria

**Figure 2:**
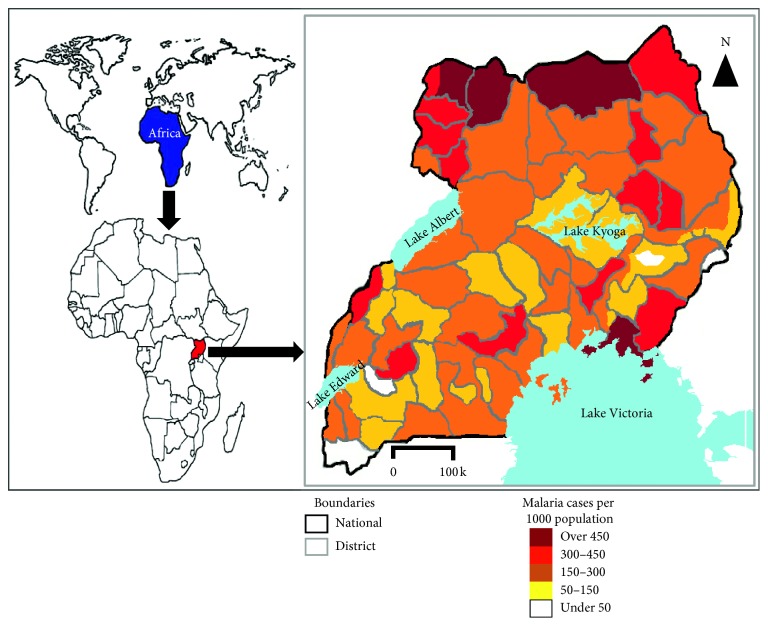
Malaria prevalence in Uganda (modified from [12]).

Malaria in Uganda is highly endemic because the climate is favorable for its consistently stable and year-round transmission in about 99% of the country, with the country's entire population being at risk for contraction [12]. The most vulnerable groups of people at great risk for malaria are expectant mothers and young children under the age of 5 years [12]. The malarial parasite, *P. falciparum*, is most commonly the cause of malaria throughout Uganda, accounting for over 90% of malaria cases. However, Betson et al. [13] have warned of the potential for the emergence of infections due to *P. malariae* and *P. ovale* spp. as well, since there is much focus on countering *P. falciparum* infections. In 2016, Larocca et al. [14] indicated that Uganda was one of the leading countries in the world with malaria incidence rate as high as 478 cases per 1,000 population per year. Specifically, overall registered death cases caused by malaria in children were between 70,000 and 100,000 annually in Uganda [14]. Tremendous effort has been made to control malaria in Uganda by the government-headed Uganda Malaria Reduction Strategic Plan and Mass Action Against Malaria. These efforts have greatly reduced the malaria burden and incidence from 272 cases per 1000 population in 2016/17 to 191 cases per 1000 population in 2017/18 [12]. Although there has been a general reduction in the incidence of malaria, studies indicate that malaria prevalence along lakes, for example, Lake Victoria, and in remote areas of the country (villages) as well as areas closer to forests are much higher, with over 450 malaria cases per 1000 population (Figure 2) [12, 13, 15]. Communities around lakeshores in Uganda have always had high prevalence of malaria among children and especially the young ones despite routine treatments [12, 16]. Through the government initiative to control malaria, the prevalence in some districts remained as low as 4.3% in 2018 [12]. Malaria control strategies including indoor residual spraying along with house to house distribution of mosquito nets treated with insecticides resulted in a remarkable reduction in malaria burdens in many parts of the country [17]. Raouf et al. [18] observed that significant reductions in the levels of malaria in Uganda cannot be sustained if the current control measures are terminated.

## 4. Mechanisms of Actions of Novel Phytochemicals in Malaria Treatment

Herbal plants are extremely rich in phytochemicals that are highly efficacious in the treatment of malaria, such as sesquiterpenes and sesquiterpene lactones, fluoroquinolones, chalcones, flavanones, phenolics, quinones, coumarins, and alkaloids (Table 1) [35, 36]. The herbal plants that are used as prophylactic measures to prevent malaria as well contain some of these compounds (Table 2). From these groups of compounds, active metabolites including quinine and artemisinin have been derived and the most successful antimalarial drugs to date have been obtained. Artemisinins from *Artemisia annua* a plant belonging to the family Asteraceae have actually been an integral part of the fight against malaria, with artemisinin-based combination therapy contributing enormously to modern day treatments [36]. They have been effective against all strains of *P. falciparum* including multi-drug-resistant ones [36, 37].

The mechanism of action of artemisinin is widely debated but the most accepted theory is that of activation of the molecule by heme, which enables it to produce free radicals that then destroy the proteins needed for parasite survival [36]. The presence of an uncommon chemical peroxide linkage bridge in artemisinin, a sesquiterpene lactone, is the most probable reason for its antimalarial effects. Cleavage of the peroxide linkage bridge in the presence of iron (II) ions (from heme) forms very reactive free radicals that undergo rapid rearrangement to form more stable carbon-centered radicals, which chemically modify the parasite and inhibit various processes within the parasite molecules, resulting in its death [36]. Artemisinin acts on primarily the trophozoite parasitic phase and prevents disease progression. It kills circulating ring-stage parasites, thus increasing the therapeutic response [37]. Mok et al. [38] suggested that artemisinin is linked to the upregulation of unfolded protein response pathways, which leads to decreased parasitic growth and development. Shandilya et al. [39] suggested that artemisinin is activated by iron, which then functionally inhibits PfATP6, a calcium pump, by terminating phosphorylation, nucleotide binding, and actuator domains, eventually leading to a functional loss of PfATP6 of the *Plasmodium* parasite and its death. A study by Mbengue et al. [40] indicated that artemisinin strongly inhibits phosphoinositide-3-kinase (PfPI3K), an enzyme important in cellular activities including growth, multiplication, differentiation, and survival in *P. falciparum.*

Cinchona tree bark, from which quinine was isolated, has been used to treat malaria since 1632 [41]. The World Health Organization listed quinine as one of the important medicines needed in a health system [42]. It is however only used to treat malaria caused by chloroquine-resistant strain of *P. falciparum* in the absence of artemisinins [43]. A popular hypothesis about the mechanism of action of quinine is based on chloroquine, another quinoline drug which is closely linked to quinine and has been comprehensively studied. Quinine inhibits the pathway of biocrystallization of hemozoin, resulting in the accumulation of the free cytotoxic heme which eventually kills the parasite [44].

Most of the plants used in the treatment of malaria in Uganda contain alkaloids greatly implicated in antiplasmodial activity (Table 3). A number of alkaloids target apicoplast, an organelle in the *Plasmodium* parasite, while others such as benzylisoquinoline alkaloids in *Cissampelos mucronata*, a plant belonging to the family Menispermaceae inhibits protein synthesis in the parasite [99].

Flavonoids in a vast number of plants used for malaria treatment in Uganda are common to plants in the family Asteraceae such as *B. longipes*, *A. conyzoides*, and *A. africana* although other herbal plants from different families including *C. roseus* in Apocynaceae and *A. zygia* and *A. nilotica* in Mimosaceae also have them as active antiplasmodial constituents (Table 3). Flavonoids exhibit great antiplasmodial activity against different strains of the malaria parasite although the mechanism of antimalarial action is not clear [99]. Some studies suggest that flavonoids impede the influx of myoinositol and L-glutamine in erythrocytes that are infected [99]. Some flavonoids increase the level of oxidation of erythrocytes and inhibit protein synthesis in malaria parasites [99]. Furthermore, flavonoids are believed to inhibit fatty acid biosynthesis (FAS II) in Plasmodium [102].

Artemisinin resistance in *P. falciparum* has been reported in Vietnam, Cambodia, Muang Lao, and Thailand. A report published in 2018 showed over 30 separate cases in Southeast Asia of artemisinin resistance [36]. In case of resistance, parasitic clearance is slowed down and gametocytemia increases, resulting in greater selective pressure on other partner drugs to which resistance increases, thereby posing a great health threat. Thus, it is very important that the discovery of other drugs with novel mechanisms of action be prioritized by extensive exploration of the huge medicinal plant resources in Africa, which have been used by locals for effective malaria treatment yet have never been scientifically investigated for their antimalarial potential. Amoa Onguéné et al. [35] emphasized that it was indeed Africa's turn to offer a new antimalarial drug to humanity since artemisinin was discovered in Asia and quinine in Latin America.

## 5. Herbs and Plant Parts Used to Manage and Treat Malaria across Communities in Uganda

**Figure 3:**
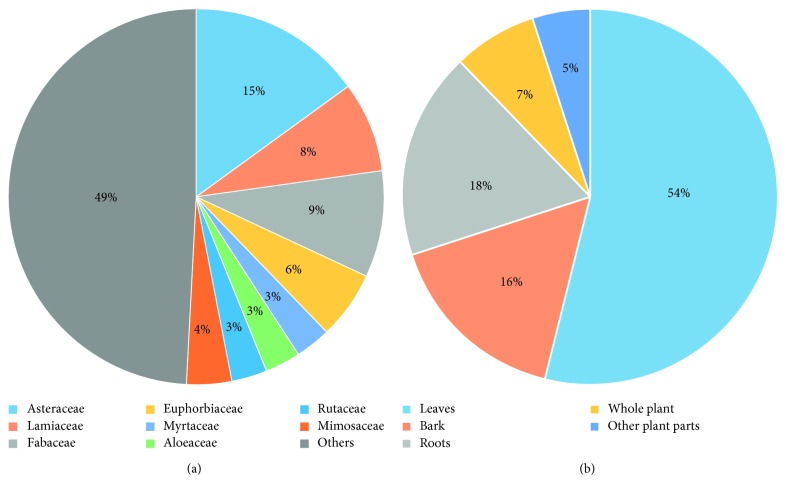
(a) Composition of plant species in each family used to treat malaria. (b) Percentage use of plant parts for treatment of malaria.

About 182 plant species from about 63 different plant families are used to treat malaria across several communities in Uganda (Table 1). Of the 63 plant families, species within the family Asteraceae are most widely used in the country to treat malaria, constituting up to 15% of all plant species used (Figure 3(a)). This is followed by species from Fabaceae (9%), Lamiaceae (8%), Euphorbiaceae (6%), and Mimosaceae (4%) families, with Myrtaceae, Aloeaceae, and Rutaceae families each contributing approximately 3% to the total number of species used for malaria treatment in Uganda (Figure 3(a)). The remaining families contribute only 49% of the total plant species used for malaria treatment (Figure 3(a)).

The plant parts greatly used to treat malaria are leaves (54.4%) followed by roots (17.4%) and bark (16%); whole plants and other plant parts are used less commonly (Figure 3(b)). A particular herbal plant is commonly used singly though some times in combination with other herbs. The most common way of use is by boiling the medicinal plant part in water and then drinking the decoction; ingestion of fresh extracts and powdered forms of the herbs is also practiced (Table 1).

Different herbal remedies are used in different communities in different parts of the country depending on the geographical distribution of the medicinal plant species, for example, *Warburgia ugandensis* is particularly used in the eastern part of Uganda. However, herbal plant species such as *Bidens pilosa L.* are spread throughout the country and thus well known for malaria treatment across the country. In a study conducted by Ssegawa and Kasenene [20], no tree species in the forests of southern Uganda were more useful than *Hallea rubrostipulata* and *Warburgia ugandensis* in the treatment of malaria. These medicinal plants are known by different local names in different parts of the country as Uganda has diverse ethnic groups, including the Luo, Baganda, Itesots, and Banyankole/Bakiga.

Among all communities in Uganda, some measures are taken to control malaria, including draining of stagnant water, clearing and burning bushes, sleeping under insecticide-treated mosquito nets, and house spraying with insecticides.

## 6. Mode of Preparation and Use of Herbs in Treatment of Malaria in Uganda

The mode of preparation and use of herbs among different communities vary depending on the nature of the herb and plant parts used for malaria treatment [10]. Most commonly, the herbal medicines are prepared as water extracts in the form of decoction and infusion or as steam baths (Table 1) [19, 23]. The herbal plant water extract is made mostly by boiling a handful of the medicinal plant parts such as leaves in a litre of water and then given to the patient to take orally (Table 1) [23]. The dose of the extract given is dependent on the age of the patient and the “strength” of the herbal medicine although occasionally the weight of the patient [19, 23]. The quantity of extract given ranges from 100 to 500 ml, 100 to 250 ml, and 1 to 3 tea or tablespoons for adults, older children, and young children below 5 years of age, respectively, between 1 and 3 times a day for about a week or until when patient has recovered [19, 25]. The extracts are mostly prepared from single herbal plants or from combination of two herbal plants, for example, a decoction of *Tamarindus indica* and *Mangifera indica* is common [25].

In some cases, the medicinal plant parts are dried then pulverized to powder and 2–5 tablespoons of the power added to water and boiled to make a decoction. Some medicinal plant parts such as bark of *M. indica* stem and roots of *V. lasiopus* and their powders are boiled for long until the water is half the initial amount [25]. The herbal plant powder can also be added to cold or hot water and stirred and then drunk as recommended [10].

Medicine for malaria treatment from a herb such as *B. pilosa* can be made by squeezing a handful of its freshly picked leaves and drinking 1–3 teaspoons of the extract a day (Table 1) [23]. Occasionally, malaria herbal medicines can be obtained by preparing different plant parts in combination, for example, an infusion can be made from fresh leaves and pounded fresh roots of *V*. *amygdalina* [25]. This is then taken orally in a recommended dose. A handful of medicinal plant parts such as leaves can be squeezed and mixed with cold or warm water for bath, for example, leaves of *B. adoensis* [25]. Some common herbs are also eaten as vegetables as a prophylactic measure against malaria while others are planted in pots around houses or burnt to drive away mosquitoes (Table 2).

## 7. Antimalarial Activities and Toxicity of Herbs Used in Uganda for Malaria Treatment

Some studies have been performed on antiplasmodial/antimalarial activities of some of the herbal plants used in Uganda to treat malaria by using various strains of malarial parasites to confirm effectiveness as malaria treatment [26, 28]. Furthermore, a broad range of phytochemicals responsible for biological activities in some of the antimalarial herbs have been isolated and identified [23]. Of the 182 plant species used in Uganda for the treatment of malaria, 112 plant species (64%) have been investigated for antimalarial activities, of which 108 plants showed positive results and only four plant species did not give positive results when tested for antimalarial activities (Table 1). For about 70 plant species (39%) that are used among different communities in Uganda for the treatment of malaria, there was no record of investigation for antimalarial activities (Table 1).

The antimalarial activity of herbal plants is due to the presence of a number of metabolically active compounds [23]. These compounds may occur in the form of alkaloids, sesquiterpenes, quinones, triterpenoids, flavonoids, quassinoids, limonoids, terpenes, chalcones, coumarins, or other miscellaneous forms [85]. The solvent of extraction largely determines the concentrations of the active metabolites in the extract. For example, methanolic extracts of the herbal plants are in general more active *in vitro* than water extracts probably due to the presence of higher amounts of more active lipophilic compounds (Table 3) [54].

The levels of activity of the antimalarial plant extracts depend on the concentration of the active antimalarial secondary metabolites [54]. For example, gedunin, a very active compound against *Plasmodium* present in leaves of *A. indica* had an IC_50_ of 0.02 *μ*g/ml against *P. falciparum*, but its concentration in the plant is in very low and thus moderate activity of its extract (Table 3) [23, 54].

The synergistic effect of the interaction of the different active secondary metabolites is a main contributing factor to the high levels of antiplasmodial activity of some of the herbal plant extracts, for example, in *A. afra*, none of the isolated flavonoids and sesquiterpenes had a high activity, yet the plant extract had an IC_50_ of 3.9 *μ*g/ml against *P. falciparum* suggesting a synergistic effect of the compounds in the extract [54]. The presence of particular active compounds in the herbal plant extracts is key in enhancing its antimalarial property. The compound 6E-geranylgeraniol-19-oic-acid a diterpene isolated from *M. pyrifolia* aqueous extract was considered responsible for its antiplasmodial activity; nitidine isolated from *Z. chalybeum* had an IC_50_ as low as 0.17 *μ*g/ml against *P. falciparum* 3D7 [10]; and pristimerin with an IC_50_ 0.5 mg/ml against *P. falciparum* was the main active ingredient in *M. senegalensis* extract, making it have a very high antiplasmodial activity [54]. The presence of a moderate amount of a minimum of two secondary metabolites in the extract could explain the efficacy of the herbal extracts for malaria treatment [10]. The pathogenic strains used may be different for different *in vitro* studies; thus, resistance of the parasite to the active metabolites could cause a variation in the level of antimalarial activity of the extracts [10]. Herbal plants with no antiplasmodial activity suggest the absence of the metabolically active compounds against the *Plasmodium* parasites in their extracts [23].  Table 4 indicates a list of herbal plants used for malaria treatment in Uganda with high antiplasmodial activities (IC_50_<5 *μ*g/ml in one of its solvent extracts or high percentage inhibition of plasmodia) that could be potentially investigated further.

Although herbs are generally considered safer when used for treatment compared to conventional drugs, some of the herbs used traditionally to treat malaria in Uganda may be efficacious, but there is a need to have them used with caution as some may be toxic (Table 4). There is a variation in degree of toxicity depending on the sensitivity of animals, tissue or cells used, type of extract, nature of the test substance, dose, and mode of administration [114]. According to Lacroix et al. [32] one third of the herbs for malaria treatment in Uganda they investigated had significant antiplasmodial activity with low toxicity. Some of the plant parts with good antiplasmodial/antimalarial activities with no or low toxicity include leaves of *A. annua,* leaves of *A. africana, S. pinnata* whole plant, leaves of *C. papaya*, and leaves of *F. virosa* amongst others (Table 4). There are however extracts of some plants used for malaria treatment with very good activity against *Plasmodium* but with high toxicity; such plant extracts include petroleum ether leaf extract of *V. amygdalina* and dichloromethane leaf extract of *M. pyrifolia* (Table 4) [32, 55]. *Clerodendrum rotundifolium* is on those plants that have very good antimalarial/antiplasmodial activities but have not been investigated for their toxicity (Table 4) [33].

## 8. Traditional Health Care Practice and Policy Framework in Uganda

The health care system of Uganda consists of the public, private-profit oriented, and private-nonprofit oriented sectors. There is quite a large sector of informal health care including traditional medicine practitioners, drug shops, medicine vendors, and complementary and alternative practitioners. The contribution of traditional health practitioners to Uganda's health care system was not valued until lately [115]. The negative perspective could be traced back to the colonial times when culture including use of traditional medicine such as herbs for treatment was considered primitive and so discouraged [115]. Efforts are now being made to promote the use of traditional medicine since the government has realized that traditional health practitioners are key contributors to its primary health care system [115]. The Ministry of Health created a public-private partnership with the traditional health practitioners following a recommendation that they be brought into the mainstream health system [115, 116].

A policy on Traditional and Complementary Medicine was created to regulate traditional medicine practice focusing on research and development while emphasizing the propagation, protection, and sustainable use of medicinal plant resources [115, 116]. For collaboration between the mainstream health care sector and traditional health practitioners, the Ministry of Health submitted a bill for the creation of the National Council of Indigenous and Complementary Medicine Practitioners, a semiautonomous body that shall as well protect their intellectual property rights [115, 116].

The National Drug Authority (NDA) is a body that ensures quality control of all medical products including herbal medicines in Uganda under the government statute and policy of 1993 [117]. In Uganda, there is no special regulatory measure for herbal medicines in that the same laws and policies for conventional pharmaceuticals also apply to the herbal medicinal products. A policy was introduced in 2002 to have herbal medicines registered, but so far, no registration of any herbal medicine has been made [117].

Herbal medicines though vastly used in Uganda are not sufficiently regulated. A system to license and track traditional health practitioners or their products is still lacking in the country, and the efforts to have the TCM integrated in the mainstream health care system is still a long way from being realized.

## 9. Conclusion

Uganda is rich in indigenous plant resources that are used by its people to treat malaria. Communities in different regions of the country use different herbs within their geographical range, though a few common herbs are used by different communities across the country. Many herbs used for malaria treatment among several communities have not been investigated for their efficacy, and yet they could be potential sources for antimalarial remedies including drugs. Few studies have been conducted to document herbs for malaria treatment in the country, especially in the northern region. Some of the plants investigated for antimalarial/antiplasmodial activities have been found to lack efficacy, toxicity, and safety study aspects. Some plants used in the local communities had very strong antimalarial activities and could be investigated further for the identification and validation of the potential therapeutic antimalarial compounds. This review is critical in that it clearly highlights herbal plants documented in Uganda for malaria treatment but have never been investigated for their antimalarial potential, thus providing guidance for further research on potential natural plant resources that could be sources of novel compounds with therapeutic properties for the treatment of malaria.

## Figures and Tables

**Table 1:** Herbs used in the treatment of malaria in Uganda.

Plant family	Scientific name	Local name	Part used	Growth form	Mode of preparation	Dose and mode of administration for malaria	Status of antimalarial/antiplasmodial activity investigation	Other ailments treated	Reference(s)
Acanthaceae	*Justicia betonica L.*	Nalongo/quinine	Leaves/whole plant	Herb	Decoction	About 120 ml every 8 hours for a week	Investigated	Diabetes, yellow fever, diarrhea	[10, 19]
	*Justicia anselliana* (Nees) T. Anderson	Kwiniini omuganda	Leaves/twig	Herb	Decoction	Orally taken, dose not specified	No record		[20]
	*Monechma subsessile* C. B. Clarke	Erazi	Leaves		Decoction	Orally taken, dose not specified	No record	Abdominal pain	[19]
	*Thunbergia alata* Sims	Kasaamusaamu/ntudde buleku	Leaves/whole plant	Climber	Decoction	About 120 ml every 8 hours for a week	No record	False teeth	[8, 10]
Alliaceae	*Allium cepa L.*	Katungulu	Bulb	Herb			No record		[21]
Aloeaceae	*Aloe dawei* A. Berger (wild/cultivated)	Kigagi	Leaves	Herb	Decoction	A glassful once a day for 7 days	Investigated	Candida	[10]
	*Aloe kedongensis* (wild)	Kigagi	Leaves	Herb	Decoction	Orally taken, dose not specified	Investigated		[19, 22]
	*Aloe volkensii* (cultivated)	Kigagi	Leaves	Herb	Decoction/infusion	Orally taken, dose not specified	No record		[19]
	*Aloe ferox* Mill	Kigagi	Leaves	Herb	Decoction	Orally taken, dose not specified	Investigated	Wounds, digestive disorders, rheumatic arthritis	[18, 19]
	*Aloe lateritia* (wild)	Kigagi	Leaves/root	Herb	Decoction	Orally taken, dose not specified	No record		[19]
Amaranthaceae	*Amaranthus hybridus L.*	Bbuga	Leaves	Herb	Decoction	Half a glass every 24 hours for 7 days	No record		[10]
Anacardiaceae	*Mangifera indica L.*	Muyembe gwakona	Leaves/bark	Tree	Decoction	4 and 3 teaspoons after every 8 hours for adults and children, respectively, for a week	Investigated	Diarrhea, dysentery, body pain, venereal diseases, cough, syphilis	[10, 23]
	*Rhus natalensis* Bernh. Ex Krauss	Omesheshe	Leaves	Shrub	Decoction	Orally taken, dose not specified	Investigated		[24]
	*Rhus vulgaris* Meikle	Kakwasokwaso/tebudda	Leaves	Shrub	Decoction	Half a glass every 8 hours for 7 days	No record	Skin rush, erectile dysfunction	[10]
Apiaceae	*Heteromorpha trifoliata* Eckl. & Zeyh.	Omumemena	Leaves/roots	Herb	Decoction	Orally taken, dose not specified	No record		[19]
	*Centella asiatica* (L.) Urb.	Kabo Kabakyala/mbutamu	Leaves/whole plant	Herb	Decoction	4 teaspoons thrice a day for 4 days	Investigated		[10]
Apocynaceae	*Alstonia boonei* De Wild.	Mubajangalabi	Bark	Tree	Decoction	Orally taken, dose not specified	Investigated		[8]
	*Carissa edulis* (Forssk.) Vahl	Muyunza, ekamuriei	Roots	Herb	Decoction	Orally taken, dose not specified	Investigated	Epilepsy, fever, cough, syphilis, measles, dysentery	[21, 23]
	*Carissa spinarum* Lodd. ex A. DC.	Omuyonza	Roots		Decoction	Orally taken, dose not specified	Investigated		[19]
	*Catharanthus roseus* G. Don	Sekagya	Leaves	Herb	Decoction	About 120 ml every 8 hours for a week	Investigated		[10]
Araceae	*Culcasia faleifolia* Engl.	Ntangawuzi yomukibira	Roots	Herb	Decoction	About 120 ml once a day for a week	No record		[10]
Aristolochiaceae	*Aristolochia elegans* Mast.	Musuja welaba/nakasero	Seeds/sap	Vine	Steeped in water and drunk	A glassful once a day	Investigated	Abdominal pain, East coast fever	[8, 19]
	*Aristolochia tomentosa* Sims.	Kankapu	Stem	Climber	Infusion	Oral, dose not specified	No record	Wounds, skin diseases, snake bites	[23]
Asclepiadaceae	*Gomphocarpus physocarpus* E. Mey.	Kafumbo	Leaves	Herb	Decoction	Half a glass daily for a week	No record		[10]
Asphodelaceae	*Aloe vera* (L.) Burm. f.	Kigagi/alovera	Leaves	Herb	Decoction	1 teaspoon and 1 tablespoon 3 times a day for children and adults, respectively, for a week	Investigated	Stomach ache	[8, 25]
Asteraceae	*Ageratum conyzoides L.*	Namirembe	Whole plant/leaves	Herb	Decoction	A glassful thrice a day for 7 days	Investigated	Worms, weakness in pregnancy	[8, 10]
	*Artemisia annua L.*	Sweet anne	Leaves	Herb	Decoction	Oral, dose not specified	Investigated	Fever	[19]
	*Artemisia afra* Jacq. ex Willd	Pasile	Leaves	Herb	Infusion	Oral, dose not specified	Investigated	Fever	[10]
	*Aspilia africana* (Pers.) C. D. Adams	Makayi, ekarwe	Whole plant/leaves/roots	Herb	Decoction	8 teaspoons 3 times a day for a week	Investigated	Abdominal aches, measles, diarrhea, wounds, induction of appetite	[10, 19]
	*Baccharoides adoensis* (Sch. Bip. ex Walp.) H. Rob.	Okellokello	Leaves	Shrub	Decoction	1 teaspoon and 1 tablespoon 3 times a day for children and adults, respectively, for a week; bath-leaves squeezed and added to bathing water	Investigated	Flu, skin rush, ear infections	[25, 26]
	*Bidens grantii* Sherff	Ehongwa	Leaves, flower	Herb	Decoction	Oral, dose not specified	No record	Pregnancy disorders, prehepatic jaundice	[19]
	*Bidens pilosa L.*	Sere/labika	Whole plant/leaves	Herb	Decoction/fresh leaf extract	4 teaspoons thrice a day for 4 days	Investigated	Diarrhea, wounds	[10, 23]
	*Bothriocline longipes* N. E. Br.	Ekyogayanja	Leaves		Decoction	Oral, dose not specified	Investigated	Fever, ague, paludism	[19, 24]
	*Conyza bonariensis* (*L.*)	Ndasha	Leaves		Decoction	Oral, dose not specified	No record	Stomach ache, body pain, anemia, respiratory problems	[19]
	*Conyza floribunda* H. B. K.	Kafumbe	Leaves	Herb	Decoction	About 120 ml once a day for a week	No record	Headache	[10]
	*Conyza sumatrensis* (Retz.) E. H. Walker	Kati kati	Leaves	Herb			No record	Wounds, sore throat, ringworms	[21, 27]
	*Crassocephalum vitellinum*	Kitonto	Leaves	Herb	Honey added to decoction	2 teaspoons thrice a day for 7 days	Investigated		[10, 19]
	*Emilia javanica* (Burm. F.) C. B. Rob.	Nakate	Whole plant	Herb	Decoction	Half a glass once a day for a week	No record		[10]
	*Guizotia scabra* Chiov.	Ekiterankuba	Leaves		Decoction	Oral, dose not specified	Investigated	Stomach ache, HIV/AIDS opportunistic infections	[19]
	*Gynura scandens* O. Hoffm.	Ekizimya-muriro	Leaves		Decoction	Oral, dose not specified	No record	Febrile convulsions	[19]
	*Melanthera scandens* (Schumach. & Thonn.) Roberty	Makaayi	Leaves	Herb	Decoction	Oral, dose not specified	Investigated	Stomach ache, body odour, yellow fever	[8]
	*Pluchea ovalis* DC.	Omuneera	Leaves		Decoction	Oral, dose not specified	No record		[19]
	*Microglossa pyrifolia* (Lam.)O. Ktze	Kafugankande	Whole plant/leaves/roots	Herb	Decoction	Half a glass thrice a day for a week	Investigated	Cough, abdominal disorders, chest pain	[10, 19, 28]
	*Schkuhria pinnata* (Lam.)	Apunait	Leaves	Herb	Infusion	1 teaspoon and 1 tablespoon 3 times a day for children and adults, respectively, for a week	Investigated	Wounds, skin diseases, diabetes, ear infections, wounds	[23, 25]
	*Sigesbeckia orientalis L.*	Kyaryaho	Roots		Decoction	Oral, dose not specified	No record	Wounds, stomach ache	[19]
	*Solanecio mannii* (Hook. f.) C. Jeffrey	Omusununu	Leaves		Decoction	Oral, dose not specified	Investigated	Fever, indigestion	[19]
	*Sonchus oleraceus L.*	Entahutara	Leaves		Decoction	Oral, dose not specified	No record	Stomach ache, scars, anemia, diarrhea	[8, 19]
	*Tagetes minuta L.*	Kawunyira	Whole plant/leaves	Herb	Decoction	Half a glass thrice a day for a week	Investigated	Flu, headache, convulsions	[10]
	*Tithonia diversifolia* A. Gray	Kimyula	Leaves	Herb	Decoction	Half a glass thrice a day for a week	Investigated	Diabetes, abdominal pain	[10, 19, 25]
	*Vernonia adoensis* Sch. Bip. ex Walp.	Nyakajuma	Leaves/flowers		Decoction	Oral, dose not specified	Investigated	Diarrhea, dizziness	[19]
	*Vernonia amygdalina* Delile	Mululuza/labwori	Whole plant/roots	Shrub	Decoction	Half a glass 2 times a day for 5 days	Investigated	Headache, stomach ache, burns, baths	[8, 10, 19, 20]
	*Vernonia cinerea* (L.) Less.	Kayayana	Bark	Tree	Decoction	Half a glass thrice a day for a week	Investigated	Fever, vomiting, inflammation	[10]
	*Vernonia lasiopus* O. Hoffm.	Kaluluza kasajja	Roots/leaves	Shrub	Fresh leaf extract/root decoction	2 teaspoons thrice a day for 7 days	Investigated	Abdominal pain, cough, migraine headache, delayed delivery	[8, 10, 19, 20]
Bignoniaceae	*Markhamia lutea* (Benth.) K. Schum.	Musambya/muzanganda	Roots	Tree	Decoction	A glassful once a day for 7 days	Investigated	Cough, diarrhea	[8, 10, 19]
	*Spathodea campanulata* Buch. -Harm. ex DC.	Kifabakazi	Bark	Tree	Decoction	Half a glass 3 times a day for 5 days	Investigated	Increased vaginal fluid, skin infection, infertility, hernia	[8, 10]
Caesalpiniaceae	*Cassia didymobotrya* Fres.	Mukyula	Leaves	Shrub	Decoction	About 120 ml every 8 hours for a week	Investigated		[10]
	*Chamaecrista nigricans* Greene	Epeduru lo didi	Leaves	Herb	Infusion	Oral, dose not specified	No record	Labour induction, hypertension, retained placenta	[23]
	*Erythrophleum pyrifolia*	Omurama	Leaves/roots				Investigated		[24]
	*Senna spectabilis* (DC.) H. S. Irwin & Barneby	Gasiya	Leaves	Tree	Decoction	Half a glass twice a day for 5 days	Investigated		[10]
Caesalpinioideae	*Cassia hirsuta*	Kasagalansansi	Roots	Herb	Infusion		Investigated	Stomach pains	[23]
Canelliaceae	*Warbugia ugandensis* Sprague	Omukuzanume	Bark/leaves	Tree	Decoction/powder swallowed with banana	Half a glass once a day for a week	Investigated	Toothache, flu, skin diseases, asthma, stomach ache, body and muscle pain	[10, 20, 27]
Caricaceae	*Carica papaya L.*	Paapali essajja	Leaves	Tree	Decoction	Half a glass twice a day for 3 days	Investigated	Snake bite, sterility, cough, cancer, body pain, induces labour	[10, 19, 23, 25]
Celastraceae	*Maytenus senegalensis*	Echomai	Roots	Tree	Decoction	Oral, dose not specified	Investigated	Toothache, skin diseases, chest pain, wound, fever	[23]
Chenopodiaceae	*Chenopodium ambrosioides L.*	Kawuna wuna	Leaves				Investigated	Headache, epilepsy	[21]
	*Chenopodium opulifolium* Koch & Ziz	Namuvu	Leaves				No record	Oral wounds, skin rush, toothache	[8, 21]
Combretaceae	*Combretum molle* G. Don	Ndagi	Bark	Tree	Decoction	Half a glass once a day for 3 days	Investigated	Cough,	[10, 21]
Crassulaceae	*Kalanchoë densiflora* Rolfe	Kisanasana	Leaves	Herb			No record		[21]
Cucurbitaceae	*Cucurbita maxima* Lam.	Kasuunsa	Leaves	Herb	Decoction	Half a glass once a day for 7 days	Investigated	Abdominal pain	[10, 25, 27]
	*Momordica foetida* Schumach.	Orwihura	Leaves		Decoction	Oral, dose not specified	Investigated	Vomiting, baths, cough, flue, worms	[19, 26, 28]
Dracaenaceae	*Dracaena steudneri* Engl.	Kajjolyenjovu	Leaves	Herb	Decoction	Half a glass thrice a day for a week	No record	Scars, cough, syphilis, kidney stones, snake bites	[8, 10]
Ebenaceae	*Euclea latideus* Staff	Emusi	Roots	Shrub	Decoction	Oral, dose not specified	Investigated	Ringworms, swollen legs	[23]
Euphorbiaceae	*Alchornea cordifolia* (Schumach.) Mull. Arg.	Luzibaziba	Leaves	Herb	Decoction	Half a glass once a day for 7 days	Investigated	Shaking body	[8, 10]
	*Bridelia micrantha* Baill.	Katazamiti	Bark	Tree	Decoction	Half a glass thrice a day for a week	Investigated		[10]
	*Clutia abyssinica* Jaub. & Spach	Omubarama	Leaves		Decoction	Oral, dose not specified	Investigated	Fever, diarrhea	[19]
	*Croton macrostachyus* Olive.	Ookota	Roots/bark	Tree	Decoction	Oral, dose not specified	Investigated	Tuberculosis, stomach ache, cough, fever, asthma	[23]
	*Fluegea virosa* (Roxb. ExWillb.)Voigt	Lukandwa/mukandula	Leaves	Shrub	Decoction	Half a glass 3 times a day for a week	Investigated	Miscarriage, chest pains, infertility in women	[8, 10, 21, 23]
	*Jatropha curcas L.*	Kirowa	Leaves	Shrub			Investigated	Tooth decay, headache, weakness in pregnancy	[21]
	*Macaranga schweinfurthii* Pax	Kyeganza	Bark	Tree	Decoction	Half a glass 3 times a day for a 5 days	No record		[10]
	*Phyllanthus (pseudo) niruri* Mull. Arg.	Nakitembe	Leaves	Shrub	Decoction	Half a glass 3 times a day for a 7 days	Investigated		[10]
	*Shirakiopsis elliptica* (Hochst.) H.–J. Esser	Musasa	Back	Tree	Decoction	Oral, dose not specified	No record		[20]
	*Tetrorchidium didymostemon* (Baill.) Pax & K. Hoffm.	Ekiziranfu	Bark		Decoction	Used as enema	No record	Jaundice, measles, gastrointestinal disorders, enema	[8, 19]
Fabaceae	*Arachis hypogea* (NC)	Ebinyobwa	Leaves		Fresh extract	Oral, dose not specified	No record		[19]
	*Cajanus cajan* (L.) Druse	Entondaigwa	Leaves	Shrub	Fresh extract	100 ml once a day for a week	Investigated	Diarrhea, body pain	[27]
	*Crotalaria agatiflora* Schweinf.	Kijjebejebbe	Whole shoot	Shrub	Fresh extract	Daily bath	No record	High blood pressure	[10]
	*Crotalaria ochroleuca* G. Don	Alayo	Leaves	Herb	Fresh extract	1 teaspoon and 1 tablespoon 3 times a day for children and adults, respectively, for a week	No record	Stomach ache	[28]
	*Entada abyssinica* Steud. ex A. Rich.	Mwolola	Leaves	Tree	Decoction		Investigated	Oral wounds, body weakness, wounds, skin infections	[8, 20, 26]
	*Entada africana* Guill. & Perr.	Mwolola	Bark	Tree	Decoction	4 and 3 teaspoons after every 8 hours for adults and children, respectively, for a week	Investigated		[10]
	*Erythrina abyssinica* Lam.	Girikiti/lacoro	Bark	Tree	Decoction	Half a glass 3 times a day for a 5 days	Investigated	Fever, leprosy, burns, tuberculosis, toothache, syphilis	[10, 23]
	*Erythrina excelsa* Bak.	Bajjangala	Bark	Tree	Decoction	Half a glass 3 times a day for a week	No record	Wounds, candida	[10]
	*Indigofera arrecta* Hochst. Ex A. Rich	Omushoroza	Roots/bark				No record	Abdominal pain	[19]
	*Indigofera congesta* Baker	Namasumi	Twig	Herb	Infusion	Oral, dose not specified	No record		[8, 20]
	*Indigofera emerginella* Steud. ex A. Rich	Omunyazabashumba	Leaves/roots	Shrub	Decoction	Oral, dose not specified	Investigated	Cough	[19]
	*Macrotyloma axillare* Verdc.	Akihabukuru	Leaves				No record	Impotence, dizziness	[19]
	*Pseudarthria hookeri* Wight & Arn	Omukongorani/kikakala	Leaves/whole plant	Herb	Decoction	One teaspoon thrice a day for 4 days	No record	Fever	[19, 20, 25, 29]
	*Rhynchosia viscosa* DC	Omutegansi	Flower				No record	Labour induction	[19]
	*Senna absus* (L.) Roxb.	Mucuula	Shrub	Leaves	Fresh extract	Oral, dose not specified	No record	Prolonged embryo in uterus	[8]
	*Senna didymobotrya* (Fresen.) H. S. Irwin & Barneby	Omugabagaba/kivumuzi	Herb	Leaves, twig	Decoction	Oral, dose not specified	Investigated	Change of sex of child	[8, 19, 20, 29]
	*Senna siamea* (Lam.) H. S. Irwin & Barneby	Garcia	Roots	Tree	Fresh extract	A cupful (500 ml) once a day for 3 days	Investigated	Abdominal pain, sore throat	[25, 27]
	*Tamarindus indica L.*	Cwaa/nkoge	Bark	Tree	Decoction	Oral, dose not specified	Investigated	Convulsions, fever	[8, 21]
Flacourtiaceae	*Ocoba spinosa* Forssk	Ekalepulepu	Roots	Herb	Decoction	Oral, dose not specified	No record	Syphilis, skin problems, wounds, headache, impotence, stomach ache	[23]
	*Trimeria bakeri* Gilg.	Omwatanshare	Leaves	Shrub	Decoction	Oral, dose not specified	Investigated		[24]
Hypericaceae	*Harungana madagascariensis* Lam.	Mukaabiransiko/mulirira	Bark	Tree	Decoction	2 tablespoons thrice a day for 3 days	Investigated	Yellow fever	[8, 10]
Labiatae	*Hyptis pectinata* Poir.	Bongoloza	Whole plant	Herb	Decoction	Oral, dose not specified	No record		[20, 29]
Lamiaceae	*Aeolanthus repens* Oliv.	Ntulagi	Leaves	Herb	Decoction	Quarter a glass thrice a day for 3 days	No record		[10]
	*Ajuga remota* Benth.	Kitinwa	Leaves	Herb	Decoction	Half a glass once a day for a week	Investigated	Stomach ache	[10]
	*Clerodendrum myricoides* R. Br.	Kikonge	Leaves	Shrub	Decoction	Half a glass daily for a week	Investigated	Syphilis, intestinal problems, induction of labour	[10, 28]
	*Clerodendrum rotundifolium* Oliv.	Kisekeseke	Roots/leaves	Shrub	Fresh leaf extract/root decoction	Half a glass daily for a 5 days	Investigated	Diabetes	[10]
	*Hoslundia opposita* Vahl.	Kamunye	Leaves	Herb	Decoction	Half a glass 3 times a day for a week; bath	Investigated	Ulcers	[8, 10, 25]
	*Leonotis nepetifolia* Schimp. exBenth	Kifumufumu	Whole plant	Herb	Decoction	A glassful thrice a day for 3 days	Investigated	Headache	[10, 21]
	*Ocimum basilicum*	Emopim	Leaves	Herb	Infusion	Half a glass 3 times a day for a week	Investigated	Fever, eye cataract	[23, 27]
	*Ocimum gratissimum* Willd.	Mujaaja	Leaves	Herb	Decoction	Half a glass 3 times a day for 5 days	Investigated	Wounds, ear infections, chest pain	[10, 21]
	*Ocimum lamiifolium* Hochst.	Omwenyi	Leaves		Decoction	Half a glass 3 times a day for a week	Investigated	Abdominal pain	[19]
	*Plectranthus barbatus*	Ebiriri omutano	Whole plant/leaves, roots/stem	Herb	Infusion	Oral, dose not specified	Investigated	Fever, heart disease, snake bite	[10, 23]
	*Plectranthus caninus* Roth	Kibwankulata	Leaves	Herb	Decoction	4 and 2 teaspoons thrice a day for adults and children, respectively, for a week	No record		[10]
	*Plectranthus cf. forskohlii*	Ekizera	Leaves		Decoction	Oral, dose not specified	No record		[19]
	*Rosmarinus officinalis L.*	Rosemary	Leaves	Herb	Decoction	Half a glass twice a day for 5 days	Investigated	Chest pain	[10]
	*Tetradenia riparia* (Hochst.) Codd	Kyewamala	Leaves	Herb	Decoction	One teaspoon twice a day for a week	Investigated		[10]
Lauranceae	*Persea americana* Mill.	Ovakedo	Leaves	Tree	Decoction	Oral, dose not specified	Investigated	Fungal and bacterial infection, high blood pressure, intestinal worms and parasites	[23]
Loranthaceae	*Tapinanthus constrictiflorus* (Engl.) Danser	Enzirugaze	Leaves	Herb	Decoction	A glass daily for 7 days	No record		[10]
Malvaceae	*Hibiscus surattensis L.*	Nantayitwako musota	Leaves	Shrub	Decoction	Half a glass thrice a day for 7 days	No record	High blood pressure	[10]
Meliaceae	*Azadirachta indica* A. Juss.	Neem	Leaves	Tree	Decoction	About 120 ml once a day for 7 days	Investigated	Dental decay/ache, yellow fever, cough, skin diseases, diabetes, nausea	[10, 19, 23, 25]
	*Carapa grandiflora* Sprague	Omukeete	Leaves/bark	Tree	Decoction	Half a glass twice a day for 7 days	No record		[10]
	*Melia azedarach*	Elira	Leaves	Tree	Decoction	Oral, dose not specified	Investigated	Fever, skin disease, itching wounds, parasitic worms	[23]
Menispermaceae	*Cissampelos mucronata* A. Rich.	Kavawala	Leaves/whole plant	Herb	Decoction	Half a glass twice a day for 5 days	Investigated		[10]
Mimosaceae	*Acacia hockii* De willd	Ekisim	Roots	Tree	Decoction	Oral, dose not specified	No record	Diarrhea, syphilis, dysentery	[23, 30]
	*Acacia nilotica*						Investigated		[31]
	*Acacia sieberiana*	Etiriri	Roots	Tree	Decoction	Oral, dose not specified	No record	Dysentery, epilepsy, cough	[21, 23]
	*Albizia coriaria* Welw.	Lugavu	Bark	Tree	Decoction	1 and 3 teaspoons thrice a day for children and adults, respectively, for a week.	Investigated	Skin diseases, diarrhea	[10]
	*Albizia grandibracteata* Taube	Nongo	Bark	Tree	Decoction	Half a glass once a day for a week	Investigated	Yellow fever, anemia, fungal infections of scalp	[8, 10, 32]
	*Albizia zygia* (DC.) Macbr.	Mulongo	Bark	Tree			Investigated		[21]
	*Newtonia buchananii* (Baker) Gilb. & Perr.	Mpewere	Bark	Tree	Dried, powdered, added to boiling water	Half a glass once a day for a week	No record		[10]
Moraceae	*Antiaris toxicaria* Lesch.	Kirundu	Bark	Tree	Decoction	Half a glass once a day for a week	Investigated	Weakness in pregnancy, headache	[8, 10]
	*Ficus natalensis* Hochst			Tree			Investigated	Gonorrhea	[8, 33]
	*Ficus saussureana* DC.	Muwo	Bark	Tree	Decoction	Half a glass thrice a day for 7 days	No record		[10]
	*Milicia excels* (Welw.) C. C. Berg.	Mivule	Bark	Tree	Decoction	Half a glass thrice a day for 7 days	Investigated	Burns, fresh cuts, skin rush	[8, 10]
Moringaceae	*Moringa oleifera* Lam	Moringa	Leaves/roots	Tree	Decoction/chewed raw	A glassful thrice a day for 7 days; a handful of fresh leaves chewed 3 times for 4 days	Investigated	Joint pains	[21, 25]
Musaceae	*Musa paradisiaca* (NC)	Kabalagala	Leaves	Herb	Decoction	Oral, dose not specified	Investigated	Jaundice, prolonged embryo in uterus	[19]
Myricaceae	*Myrica kandtiana Engl.* (NC)	Omujeeje	Leaves		Decoction	Oral, dose not specified	No record	Vomiting, diarrhea	[19]
Myristicaceae	*Pycnanthus angolensis* (Welw.)Warb.	Lunaba	Leaves	Tree	Decoction	Half a glass a day	Investigated		[10]
Myrsinaceae	*Maesa lanceolata* Forssk.	Kiwondowondo	Leaves	Shrub	Decoction	Half a glass thrice a day for 7 days	Investigated	Febrile convulsions	[10, 19, 24]
Myrtaceae	*Eucalyptus grandis* Maiden.	Kalitunsi	Leaves	Tree	Decoction	Half a glass a day	No record	Cough	[8, 10]
	*Psidium guajava L.*	Mupeera	Leaves	Tree	Decoction	Half a glass thrice a day for a week	Investigated	Bloody diarrhea, typhoid, wounds, cough	[10, 23]
	*Syzygium cordatum* Hochst.	Mugeege	Bark	Tree	Decoction	Oral, dose not specified	Investigated	Dry cough, skin rush, wounds	[8, 10, 20, 29]
	*Syzygium cumini* (L.) Skeels	Jambula	Leaves	Tree	Decoction	Half a glass thrice a day for a week	Investigated	Cough	[32]
	*Syzygium guineense* (Willd.) DC.	Kalunginsanvu	Bark	Tree	Decoction	Oral, dose not specified	Investigated		[20]
Papillionaceae	*Butyrospermuum paradoxum*	Ekunguri	Roots	Tree	Decoction	Oral, dose not specified	No record	Labour pains, headaches	[23]
	*Ormocarpum trachycarpum*	Ederut	Roots	Shrub	Decoction	Oral, dose not specified	No record	Pneumonia, snake bite	[23]
Passifloraceae	*Passiflora edulis* Sims	Akatunda	Leaves	Herb	Fresh extract	Oral, dose not specified	No record	Diarrhea, cough	[19]
Pittosporaceae	*Pittosporum brachcalya*	Not defined	Not defined	Shrub			No record		[34]
	*Pittosporum mannii* Hook. f. Subsp. ripicola (J. Leon)Cuf.	Mubajjankon	Leaves	Shrub	Infusion/decoction	Half a glass a day for a week	No record		[10]
Poaceae	*Cymbopogon citratus* Stapf.	Kisubi	Leaves	Grass	Decoction	120 ml every after 8 hours for a week	Investigated	Dental caries, influenza, cough, cancer, indigestion, fever	[10, 19, 23]
	*Digitaria scalarum* Chiov.	Lumbugu	Leaves	Grass	Decoction	120 ml every after 8 hours for a week	No record		[10]
	*Imperata cylindrical* (L.) Beauv. var. africana (Anderss.) C. E. Hubbard	Lusenke	Roots	Grass	Dried, powdered, added boiling water/decoction	120 ml once a day for a week	No record	Abdominal pain	[10]
	*Zea mays L.*	Luyange lwakasoli	Flowers/husks	Cereal grass	Decoction	120 ml every after 8 hours for a week	Investigated	Boosts immunity	[10]
Polygalaceae	*Securidaca longipedunculata* Fresen.	Eliloi	Roots	Shrub	Decoction	Oral, dose not specified	Investigated	Skin diseases, measles, cough, hernia, diarrhea	[23]
	*Maesopsis eminii* Engl.	Musizi	Bark	Tree	Decoction	Half a glass thrice a day for a week	No record		[10]
Portulacaceae	*Talinum portulacifolium (Forssk.) Asch. ex Schweinf.*	Mpozia	Leaves	Herb		Oral, dose not specified	No record		[21]
Rosaceae	*Prunus africana* (Hook. f.) Kalkman	Ntaseesa or Ngwabuzito	Bark	Tree	Decoction	2 and 3 teaspoons thrice a day for children and adults, respectively, for a week	Investigated	Fainting, cancer	[8, 10]
	*Rubus steudneri* schweinf.	Nkenene	Leaves	Herb	Decoction	Half a glass once a day for a week	No record		[10]
Rubiaceae	*Coffea canephora* Froehner	Mwanyi	Leaves	Shrub	Decoction	Oral, dose not specified	No record		[21]
	*Hallea rubrostipulata* (K. Schum.) J.-F. Leroy	Muziku	Bark	Tree	Decoction	Oral, dose not specified	Investigated		[20]
	*Pentas longiflora* Oliv.	Ishagara	Leaves		Decoction	Oral, dose not specified	Investigated	Fever	[19]
	*Vangueria apiculata* K. Schum.	Matugunda	Bark	Shrub	Decoction	2 and 3 teaspoons thrice a day for children and adults, respectively, for a week	No record		[10]
Rutaceae	*Citrus reticulata*	Omuqugwa	Roots	Tree	Decoction	Oral, dose not specified	Investigated	Weight loss induction, cancer, skin diseases	[23]
	*Citrus sinensis*	Omucungwa/cungwa	Roots	Tree	Decoction	Oral, dose not specified	Investigated	Vomiting, cough, diabetes	[21, 23, 25]
	*Teclea nobilis* Delile	Omuzo	Aerial parts		Decoction	Oral, dose not specified	Investigated	Body cleanser	[32]
	*Toddalia asiatica* Baill.	Kawule	Roots	Climber	Decoction	Half a glass thrice a day for a week	Investigated	Cough, abdominal pain	[10, 19, 24]
	*Zanthoxylum chalybeum* Engl.	Ntale ya ddungu	Roots	Tree	Decoction	Oral, dose not specified	Investigated	Body swellings, stomach ache, cough, fever, chest pain	[10, 23, 28]
	*Zanthoxyllum leprieurii* Guill. & Perr.	Mutatembwa/munyenye	Bark	Tree	Decoction drunk	Half a glass thrice a day for a week	No record		[10]
Salicaceae	*Trimeria grandifolia* ssp. tropica (Hochst.) Warb.	Omwatanshare	Leaves		Decoction	Oral, dose not specified	Investigated		[19]
Sapindaceae	*Blighia unijugata* Baker	Nkuzanyana	Bark	Tree	Decoction drunk	Half a glass twice a day for a week	Investigated	Wounds, vomiting, skin diseases, fibroids, cervical cancer	[8, 10]
Sapotaceae	*Manilkara obovata* (Sabine & G. Don)	Nkunya	Bark	Tree	Decoction	Oral, dose not specified	No record		[20]
Scrophulariaceae	*Sopubia ramosa* (Hochst.) Hochst.	Kakulunkanyi	Whole plant	Herb	Decoction	Oral, dose not specified	No record		[20]
Simaroubaceae	*Harrisonia abyssinica* Olive.	Ekeroi	Roots/leaves	Shrub	Decoction	Oral, dose not specified	Investigated	Fever, wounds, syphilis, snake bite, abdominal pain	[23]
Solanaceae	*Datura stramonium* Thunb.	Amadudu	Leaves	Herb	Decoction drunk	Half a glass thrice a day for a week	No record	Ulcers, stomach ache, chest pain	[10]
	*Physalis peruviana L.*	Ntuntunu	Leaves	Herb	Decoction drunk	Half a glass 3 times a day for a week	No record	Vomiting, febrile convulsions, fainting	[8, 10, 19]
	*Solanum nigrum L.*	Nsugga	Leaves	Herb	Decoction drunk	Half a glass 3 times a day for a week	Investigated	Ear infection, headache, epilepsy, STI, diarrhea	[8, 10]
Tiliaceae	*Trumfetta rhomboidea* Jacq.	Musombankoko	Roots	Shrub	Decoction drunk	Half a glass once a day for a week	No record		[10]
Ulmaceae	*Celtis africana L.*	Akasisa	Leaves	Tree	Decoction drunk	Half a glass a day for a week	Investigated		[10]
Umbelliferae	*Steganotania araliacea* Hoeshst	Ematule	Roots/leaves	Tree	Decoction	Oral, dose not specified	No record	Measles, body swelling	[23]
Verbenaceae	*Lantana camara*	Kanpanga	Leaves	Shrub	Decoction	Oral, dose not specified	Investigated	Wounds, measles, tuberculosis, pneumonia, snake bite, chest pain	[23]
	*Lantana trifolia L.*	Omuhukye	Leaves		Decoction	Orally taken, dose not specified	Investigated	Yellow fever, ringworms, muscle pain, prolapsed rectum	[8, 19]
Zingiberaceae	*Curcuma longa L.*	Binjali	Rhizome	Herb	Fresh extract	30 ml thrice a day for 3 days	Investigated		[28]

**Table 2:** Some herbs used in malaria prevention amongst communities in Uganda.

Plant family	Plant species	Local name	Plant form	Mode of use to prevent malaria	Reference(s)
Cleomaceae	*Cleome gynandra L.*	Akeyo	Herb	Leaves are cooked and eaten as a prophylactic measure	[25]
Cucurbitaceae	*Cucurbita maxima* Duchesne	Acuga	Scrambler	Leaves cooked and pasted with groundnut then eaten	[25]
Euphorbiaceae	*Manihot esculenta* Crantz	Gwana	Herb	Tuber peelings are dried then burnt in house so that smoke repels mosquitoes	[25]
Fabaceae	*Crotalaria ochroleuca* G. Don	Alayo	Herb	Leaves are cooked and eaten as a prophylactic measure	[25]
Lamiaceae	*Ocimum forsskaolii* Benth.	Yat cola	Herb	Leaves dried and burnt so that smoke chases away mosquitoes; bath infusion to repel mosquito	[25]
	*Rosmarinus officinalis L.*	Rosemary	Herb	Leaves are cooked and eaten as a prophylactic measure; planted around the house to repel mosquitoes	[10]
Malvaceae	*Gossypium hirsutum L.*	Pama	Shrub	Cotton lint is dried and burnt so that smoke keeps away mosquitoes	[25]
Musaceae	*Musa* sp.	Labolo kwon	Shrub	Fruit peeling are dried and burnt in the house to produce smoke that keeps away mosquitoes	[25]
Myrtaceae	*Eucalptus grandis* Maiden.	Kalitunsi	Tree	Leave and branches are burnt to repel mosquitoes	[25]
Poaceae	*Cymbopogon citratus* Stapf.	Kisubi	Grass	Planted around the house to repel mosquitoes; taken in tea as a prophylactic measure	[19, 23]
Solanaceae	*Solanum americanum* Mill.	Ocuga	Herb	Leaves are cooked and eaten as a prophylactic measure	[25]

**Table 3:** Antiplasmodial/antimalarial activities of investigated plants used for malaria treatment in Uganda and their active chemical constituents.

Plant family	Scientific name	Part used	Extracting solvent	Means of traditional extraction	Report on antiplasmodial, IC_50_ (*μ*g/ml)/antimalarial activity (*Plasmodium* strain)	Active chemical constituents	Reference(s)
Acanthaceae	*Justicia betonica L.*	Shoot	Methanol	Hot water	69.6 (chloroquine sensitive, K39)	Justetonin (indole(3,2-b) quinoline alkaloid glycoside)	[20]
			Water		>100 (chloroquine sensitive, K39)		
Aloeaceae	*Aloe dawei* A. Berger (wild/cultivated)	Leaves	Ether	Cold water; mashing; hot water	Extract had anti-*P. falciparum* activity value of 7.97 (95% CI: 3.56 to 17.85) *μ*g/ml with 50% schizonts suppression per 200 WBC (EC_50_)	Anthraquinones, aloin, lectins,	[19, 45]
	*Aloe kedongensis* (wild)	Leaves	Methanol	Hot water	87.7 (chloroquine sensitive, D6); 67.8 (chloroquine resistant, W2)	Anthrone, C-glucoside homonataloin, anthraquinones, aloin, lectins	[19, 46]
	*Aloe ferox* Mill	Leaves	Dichloromethane	Water	21 (chloroquine sensitive, D10)	Mannans, polymannans, anthraquinones, aloin, lectins, anthrones	[19, 31, 47]
			Water		>100 (chloroquine sensitive, D10)		
Anacardiaceae	*Mangifera indica L.*	Leaves	Chloroform:Methanol (1 : 1)	Hot water	Inhibited growth of *P. falciparum* by 50.4% at 20 *μ*g/ml	Phenolics	[48, 49]
		Stem bark	Ethanol		>50 (chloroquine resistant, FcB1)		
	*Rhus natalensis* Bernh. Ex Krauss	Leaves	Ethanol	Hot water	6.6 (*P. falciparum*)	Triterpenoids	[24]
Apiaceae	*Centella asiatica* (L.) Urb.	Whole plant	Water	Water	58.6 (chloroquine sensitive, D6); not detected (chloroquine resistant, W2)	Phenolics and flavonoids	[50]
Apocynaceae	*Alstonia boonei* De Wild.	Stem bark	Water	Hot water	80.97% suppressive activity at 200 mg/kg (*P. berghei*) in combination with other two local herbs.	Alkaloids, triterpenoids	[51]
	*Carissa edulis* (Forssk.) Vahl	Stem bark	Dichloromethane	Mashing; hot water	33 (chloroquine sensitive, D10)	Lignan, nortrachelogenin	[52]
	*Carissa spinarum* Lodd. ex A. DC.	Root bark	Methanol	Hot water	14.5 (chloroquine sensitive, D6)	Saponins, sesquiterpenes	[53]
	*Catharanthus roseus* G. Don	Leaves	Methanol	Hot water	4.6 (chloroquine sensitive, D6); 5.3 (chloroquine resistant, W2)	Alkaloids, terpenoids, flavonoids, esquiterpenes	[54]
Aristolochiaceae	*Aristolochia elegans* Mast.	Seeds	Methanol	Water	>50 (chloroquine sensitive, 3D7); undetectable (chloroquine resistant, W2)	Sesquiterpenoids, diterpenoids, monoterpenoids, alkaloids	[19, 55]
Asphodelaceae	*Aloe vera* (L.) Burm. f.	Leaves	Water	Cold water; mashing; hot water	Antiplasmodial activity in terms of EC_50_ values 0.289 to 1.056 *μ*g/ml (chloroquine sensitive)	Aloin, anthraquinones, aloe-emodin	[56]
Asteraceae	*Ageratum conyzoides L.*	Whole plant	Methanol	Hot water	11.5 (chloroquine sensitive, D6); 12.1 (chloroquine resistant, W2)	Flavonoids	[54]
	*Artemisia annua L.*	Leaves	Water	Hot water	1.1 (chloroquine sensitive, D10); 0.9 (chloroquine resistant, W2)	Sesquiterpenes and sesquiterpene lactones including artemisinin, flavonoids such as chrysoplenol-D, eupatorin, chyrsoplenetin	[19, 57]
	*Artemisia afra* Jacq. Ex Willd	Leaves	Methanol	Hot water	9.1 (chloroquine sensitive, D6); 3.9 (chloroquine resistant, W2)	Acacetin, genkwanin, 7-methoxyacacetin	[54]
	*Aspilia africana* (Pers.) C. D. Adams	Leaves	Ethanol	Hot water	Significant chemo suppressive effect of 92.23% (400 mg/kg) on *P. berghei*	Saponins, terpenoids, alkaloids, resins, tannins, flavonoids, sterols	[19, 58]
	*Baccharoides adoensis* (Sch. Bip. ex Walp.) H. Rob.	Leaves	Petroleum ether	Hot water	4.6 (chloroquine resistant, K1)	Flavonoids	[26]
	*Aspilia africana L.*	Leaves	Dichloromethane	Hot water; mashing	8.5 (chloroquine sensitive, D10)	Flavonoids including quercetin 3,3′-dimethyl ether 7-0-*α*-L-rhamnopyranosyl-(1 ⟶ 6)-β-D-glucopyranose and quercetin 3,3′-dimethyl ether 7-0-β-D-glucopyranose	[52]
	*Bothriocline longipes* N. E. Br.	Leaves	Chloroform	Hot water	3.7 (*P. falciparum*)	5-alkylcoumarins,	[19, 24]
			Ethanol		50 (*P. falciparum*)		
	*Crassocephalum vitellinum*	Leaves	Ethyl acetate	Hot water	40.6% inhibition of *P. falciparum* at 10 μg/ml	Flavonoids	[32]
	*Guizotia scabra* Chiov.	Whole plant	Crude ethanol	Hot water	49.09% growth inhibition at 100 μg/ml (chloroquine resistant, Dd2)	Lactones, eudesmanoline	[59]
	*Melanthera scandens* (Schumach. & Thonn.) Roberty	Leaves	Chloroform	Hot water	68.83% chemo suppression activity (*P. berghei*)	Triterpenoid saponins	[60]
	*Microglossa pyrifolia* (Lam.)O. Ktze	Leaves		Hot water	<5 (both chloroquine sensitive, NF54 and resistant, FCR3)	E-phytol; 6e-geranylgeraniol-19-oic acid	[2, 28]
	*Schkuhria pinnata* (lam.)	Whole plant	Water	Hot water	22.5 (chloroquine sensitive, D6); 51.8 (chloroquine resistant, W2)	Schkuhrin I and schkuhrin II	[54]
			Methanol		1.3 (chloroquine sensitive, D6); 6.8 (chloroquine resistant, W2)		
	*Solanecio mannii* (Hook. f.) C. Jeffrey	Leaves	Methanol	Water	21.6 (chloroquine sensitive, 3D7); 26.2 (chloroquine resistant, W2)	Phytosterols, n-alkanes and N-hexacosanol,	[19, 55]
	*Tagetes minuta L.*	Leaves	Ethyl acetate	Water	61.0% inhibition of *P. falciparum* at 10 μg/ml		[32]
	*Tithonia diversifolia* A. Gray	Leaves	Methanol	Water	1.2 (chloroquine sensitive, 3D7); 1.5 (chloroquine resistant, W2)	Tagitinin C, sesquiterpene lactones	[55]
	*Vernonia adoensis* Sch. Bip. ex Walp.	Leaves	Methanol	Hot water	83.4% inhibition of parasitaemia, at 600 mg/kg (*P. berghei*)	Glycocides, glaucolides	[19, 61]
	*Vernonia amygdalina* Delile	Leaves	Methanol/dichloromethane	Hot water; cold water	2.7 (chloroquine resistant, K1)	Coumarin, sesquiterpene lactones including vernolepin, vernolin, vernolide, vernodalin and hydroxyvernodalin, steroid glucosides	[19, 26]
	*Vernonia cinerea* (L.) Less.	Whole plant	Water	Hot water	>50 (chloroquine sensitive, 3D7); 37.2 (chloroquine resistant, K1)	Sesquiterpene lactone	[62]
	*Vernonia lasiopus* O. Hoffm.	Leaves	Methanol	Mashing; hot water	44.3 (chloroquine sensitive, D6); 52.4 (chloroquine resistant, W2)	Sesquiterpene lactones, polysaccarides	[19, 54]
Bignoniaceae	*Markhamia lutea* (Benth.) K. Schum.	Leaves	Ethyl acetate	Hot water	71% inhibition of *P. falciparum* at 10 μg/ml	Phenylpropanoid glycosides, cycloartane triterpenoids	[32]
	*Spathodea campanulata* Buch.-Harm. ex DC.	Stem bark	Ethyl acetate	Water	28.9% inhibition of *P. falciparum* at 10 μg/ml	Quinone (lapachol)	[32]
Caesalpiniaceae	*Cassia didymobotrya* Fres.	Leaves	Methanol	Hot water	23.4 (chloroquine sensitive, D6); undetectable (chloroquine resistant, W2)	Alkaloids	[54]
	*Erythrophleum pyrifolia*	Leaves	Ethanol	Hot water	>50 (*P. falciparum*)		[24]
	*Senna spectabilis* (DC.) H. S. Irwin & Barneby	Leaves	Ethanol	Water	59.29% growth inhibition at 100 mg/kg body weight dose *(P. berghei)*	Piperidine alkaloids	[63]
Caesalpinioideae	*Cassia hirsuta*	Root back	Methanol	Water	32.0 (chloroquine sensitive 3D7)		[64]
Canelliaceae	*Warbugia ugandensis* Sprague	Stem back	Methanol	Hot water	6.4 (chloroquine sensitive, D6); 6.9 (chloroquine resistant, W2)	Sesquiterpenes e.g. muzigadiolide	[27, 54]
			Water		12.9 (chloroquine sensitive, D6); 15.6 (chloroquine resistant, W2)		
Caricaceae	*Carica papaya L.*	Leaves	Ethyl acetate	Hot water	2.96 (chloroquine sensitive, D10); 3.98 (chloroquine resistant, DD2)	Alkaloids, saponins, tannins, glycosides	[65]
			Methanol		10.8 (chloroquine sensitive, D10)		
Celastraceae	*Maytenus senegalensis*	Roots		Hot water	1.9 (chloroquine sensitive, D6); 2.4 (chloroquine resistant, W2)	Terpenoids, pentacyclic triterpenes e.g. pristimerin	[66]
Chenopodiaceae	*Chenopodium ambrosioides L.*	Leaves	Crude hydroalcoholic extract	Hot water	Inhibited the *P. falciparum* growth, exhibiting an IC_50_ of 25.4 μg/ml	Sesquiterpenes, monoterpenes	[67]
Combretaceae	*Combretum molle* G. Don	Stem back	Acetone	Water	8.2 (chloroquine sensitive 3D7)	Phenolics, punicalagin	[68]
Cucurbitaceae	*Cucurbita maxima* Lam.	Seeds	Crude ethanol	Hot water	50% reduction of parasitaemia levels in *P. berghei* infected mice at 500 mg/kg.	Phenols, terpenoids, alkaloids, tannins	[69]
	*Momordica foetida* Schumach.	Shoot	Water	Hot water	6.16 (chloroquine sensitive, NF54); 0.35 (chloroquine resistant, FCR3)	Saponins, alkaloid, cardiac glycosides	[28]
Ebenaceae	*Euclea latideus* Staff	Root back	Hexane	Water	38.2 (chloroquine sensitive, 3D7); 38.9 (chloroquine resistant, Dd2)	Triterpenoids lupeol, betulin, 3β-(5-hydroxyferuloyl)lup-20(30)-ene	[23]
Euphorbiaceae	*Alchornea cordifolia* (Schumach.) Mull. Arg.	Leaves	Water	Hot water	4.8 (chloroquine resistant, K1)	Phenolics including ellagic acid	[70]
	*Bridelia micrantha* Baill.	Stem bark	Methanol	Hot water	19.4 (chloroquine sensitive, D6); 14.2 (chloroquine resistant, W2)		[50]
	*Clutia abyssinica* Jaub. & Spach	Leaves	Methanol	Water	7.8 (chloroquine sensitive, D6); 11.3 (chloroquine resistant, W2)	Diterpenes	[54]
	*Croton macrostachyus* Olive.	Leaves	Chloroform	Hot water	Chemotherapeutic effect of 66–82% in malaria mouse model	Triterpenoids including lupeol	[71]
	*Fluegea virosa* (Roxb. ExWillb.)Voigt	Leaves	Water/methanol	Hot water	2 (chloroquine resistant, W2)	Bergenin	[72]
	*Jatropha curcas L.*	Leaves	Ethyl acetate	Hot water	5.1 (chloroquine sensitive, NF54); 2.4 (chloroquine resistant, K1)	Alkaloids, saponnins, glycosides, tannins	[73]
	*Phyllanthus (pseudo) niruri* Mull. Arg.		Water	Hot water	Ranged from 2.9 to 4.1 (both chloroquine sensitive, 3D7 and resistant, Dd2)	Coumarins including 1-O-galloyl-6-O-luteoyl-a-D-glucose	[74]
Fabaceae	*Cajanus cajan* (L.) Druse	Leaves	Crude ethanol	Mashing	29.0 (*P. falciparum*)	Cajachalcone;	[75]
	*Entada abyssinica* Steud. ex A. Rich.	Seeds	Methanol	Hot water	>5 (chloroquine resistant, K1)	Flavonoids, terpenoids	[26, 32]
	*Entada africana* Guill. & Perr.	Leaves	Ethanol	Hot water	26.4 (chloroquine sensitive, HB3); 28.9 (chloroquine resistant, FcM29)	Phenolics	[76]
	*Erythrina abyssinica* Lam.	Stem bark	Ethyl acetate	Hot water	83.6% inhibition of *P. falciparum* at 10 μg/ml	Chalcones (5-prenylbutein, homobutein), flavanones including 5-deoxyabyssinin II, abyssinin III and abyssinone IV	[32]
	*Indigofera emerginella* Steud. ex A. Rich	Leaves	Ethanol	Hot water	5.8 (*P. falciparum*)		[24]
	*Senna didymobotrya* (Fresen.) H. S. Irwin & Barneby	Leaves	Methanol	Hot water	>100 (chloroquine sensitive, K39)	Quinones	[20, 29]
	*Senna siamea* (Lam.) H. S. Irwin & Barneby	Leaves	Ethanol	Mashing; hot water	28.8 (chloroquine sensitive, 3D7); 48.3 (chloroquine resistant, W2)	Phenolic derivative, chrobisiamone a, anhydrobarakol	[77]
	*Tamarindus indica L.*	Stem bark	Water	Hot water	25.1% chemo suppressive activity at 10 mg/kg (*P. berghei*)	Saponins (leaves), tannins (fruits)	[78]
Flacourtiaceae	*Trimeria bakeri* Gilg.	Leaves	Petroleum ether	Hot water	3.9 (*P. falciparum*)	Triterpenoids	[24]
Hypericaceae	*Harungana madagascariensis* Lam.	Stem bark	Water	Hot water	9.64 (chloroquine resistant, K1)	Quinones including bazouanthrone, feruginin a, harunganin, harunganol a	[70]
Lamiaceae	*Ajuga remota* Benth.	Whole plant	Ethanol	Hot water	55 (chloroquine sensitive, FCA/GHA); 57 (chloroquine resistant, W2)	Ajugarin-1, ergosterol-5,8-endoperoxide, 8-O-acetylharpagide, steroids	[79]
	*Clerodendrum myricoides* R. Br.	Root bark	Methanol	Hot water	4.7 (chloroquine sensitive, D6); 8.3 (chloroquine resistant, W2)		[50, 80]
	*Clerodendrum rotundifolium* Oliv.	Leaves	Methanol	Mashing; hot water	<5 (both chloroquine sensitive, NF54 and resistant, FCR3)	Saponins, tannins	[28]
	*Hoslundia opposita* Vahl.	Leaves	Ethyl acetate	Hot water	66.2% inhibition of *P. falciparum* at 10 μg/ml	Quinones, saponins, abietane diterpenes (3-O-benzoylhosloppone)	[32]
	*Leonotis nepetifolia* Schimp. exBenth	Leaves	Ethyl acetate	Water	27.0% inhibition of *P. falciparum* at 10 μg/ml		[32]
	*Ocimum basilicum*	Leaves	Ethanol	Hot water	68.14 (chloroquine sensitive, CQ-s); 67.27 (chloroquine resistant, CQ-r)		[50, 80]
	*Ocimum gratissimum* Willd.	Leaves/twigs	Dichloromethane	Hot water	8.6 (chloroquine resistant, W2)	Flavonoids	[47, 49]
	*Ocimum lamiifolium* Hochst.	Leaves	Water	Water	Significantly suppressed parasitaemia, 22.2%, 26.8% and 35.5% at dose of 200, 400 and 600 mg·kg, respectively (*P. berghei*)		[81]
	*Plectranthus barbatus*	Leaves/stem	Dichloromethane	Hot water	No activity		[23, 47]
	*Rosmarinus officinalis L.*			Hot water	Essential oil at a concentration 15867 ng/ml had no antimalarial activity		[82]
	*Tetradenia riparia* (Hochst.) Codd	Root		Hot water	13.2 (chloroquine-sensitive, NF54)		[83]
Lauranceae	*Persea americana* Mill.	Leaves	Ethanol	Hot water	10.15 (chloroquine sensitive, 3D7); 44.94 (chloroquine resistant, W2)	Phenolics	[84]
Meliaceae	*Azadirachta indica* A. Juss.	Leaves		Hot water	17.9 (chloroquine sensitive, D6); 43.7 (chloroquine resistant, W2)	Terpenoids, isoprenoids, gedunin	[49, 66]
	*Melia azedarach*	Leaves	Methanol	Hot water	55.1 (chloroquine sensitive, 3D7); 19.1 (chloroquine resistant, W2)		[85]
Menispermaceae	*Cissampelos mucronata* A. Rich.	Root bark	Methanol	Hot water	8.8 (chloroquine sensitive, D6); 9.2 (chloroquine resistant, W2)	Benzylisoquinoline alkaloids	[80]
Mimosaceae	*Acacia nilotica*	Stem bark	Methanol	Hot water	Dose of 100 mg/kg b/w produced parasitic (*P. berghei*) inhibition 77.7%	Tannins, flavonoids, terpenes	[86]
	*Albizia coriaria* Welw.	Stem bark	Methanol	Hot water	15.2 (chloroquine sensitive, D6); 16.8 (chloroquine resistant, W2)	Triterpenoids, lupeol, lupenone	[54]
	*Albizia grandibracteata* Taube	Leaves	Ethyl acetate	Hot water	22.0% inhibition of *P. falciparum* at 10 μg/ml		[32]
	*Albizia zygia* (DC.) Macbr.	Stem bark	Methanol	Water	1.0 (chloroquine resistant, K1)	Flavonoids mainly 3′,4′,7-trihydroxyflavone	[87]
Moraceae	*Antiaris toxicaria* Lesch.	Stem bark	Ethyl acetate	Hot water	36.4% inhibition of *P. falciparum* at 10 μg/ml		[32]
	*Ficus natalensis* Hochst	Leaves	Hexane	Hot water	6.7 (*P. falciparum*)		[88]
	*Milicia excels* (Welw.) C. C. Berg.	Leaves	Ethanol	Hot water	76.7% chemo suppressive activity at 250 mg/kg/day (*P. berghei*)		[89]
Moringaceae	*Moringa oleifera* Lam	Leaves	Methanol	Mashing; hot water	9.8 (chloroquine sensitive, D6); not detected (chloroquine resistant, W2)	Flavonols	[49, 80]
Musaceae	*Musa paradisiaca* (NC)	Leaves	Ethyl acetate	Hot water	75 (chloroquine sensitive, 3D7); 100 (chloroquine resistant, Dd2)	Flavonoids	[49, 90]
Myristicaceae	*Pycnanthus angolensis* (Welw.)Warb.	Leaves	50% ethanol	Hot water	>1000 (chloroquine sensitive, 3D7)	Talaumidin	[91]
Myrsinaceae	*Maesa lanceolata* Forssk.	Twig	Dichloromethane:Methanol (1 : 1)	Hot water	5.9 (chloroquine sensitive, D10)	Lanciaquinones, 2,5, dihydroxy-3-(nonadec-14-enyl)-1,4-benzoquinone	[24, 52, 55]
Myrtaceae	*Psidium guajava L.*	Stem back	Water	Hot water	10–20 (chloroquine sensitive, D10)	Phenols, flavonoids, carotenoids, terpenoids	[49, 92]
	*Syzygium cordatum* Hochst.	Twig	Dichloromethane:Methanol (1 : 1)	Hot water	14.7 (chloroquine sensitive, D10)		[55]
	*Syzygium cumini* (L.) Skeels	Stem back		Hot water	0.25 to 27.1 (chloroquine-resistant strains)		[93]
	*Syzygium guineense* (Willd.) DC.	Leaves	Crude ethanol	Hot water	49.09% chemo suppression at 400 mg/kg (*P. berghei*)		[94]
Poaceae	*Cymbopogon citratus* Stapf.	Whole plant		Hot water	99.89% suppression of parasitaemia at 1600 mg/kg	Flavonoids	[20, 49, 95]
	*Zea mays L.*	Husks	Ethyl acetate	Hot water	9.3 (chloroquine sensitive, 3D7); 3.7 (chloroquine resistant, INDO)	Alkaloids, flavonoids and triterpenoids	[96]
Polygalaceae	*Securidaca longipedunculata* Fresen.	Leaves	Dichloromethane	Hot water	6.9 (chloroquine sensitive, D10)	Saponins, flavonoids, alkaloids, steroids	[92]
Rosaceae	*Prunus africana* (Hook. f.) Kalkman	Stem bark	Methanol	Hot water	17.3 (chloroquine sensitive, D6); not detected (chloroquine resistant, W2)	Terpenoids	[54]
Rubiaceae	*Hallea rubrostipulata* (K. Schum.) J.-F. Leroy	Root	Ethanol	Water	100 μg/ml extract had 65.54% growth inhibition (chloroquine resistant, Dd2)	Alkaloids	[59]
	*Pentas longiflora* Oliv.	Root	Methanol	Hot water	0.99 (chloroquine sensitive, D6); 0.93 (chloroquine resistant, W2)	Pyranonaphthoquinones, pentalongin (1) and psychorubrin (2), naphthalene derivative mollugin (3)	[97]
Rutaceae	*Citrus reticulata*	Seeds (isolimonexic acid methyl ether)		Hot water	<4.76 (both chloroquine sensitive, D6 and resistant, W2)	Limonin, isolimonexic acid methyl ether, ichangin, deacetylnomilin, obacunone	[98]
	*Citrus sinensis*		70% ethanol	Hot water	53.27% suppression of parasitaemia at 700 mg/kg	Tannins, alkaloids, saponins, flavonoids	[20, 24, 99]
	*Teclea nobilis* Delile	Bark	Ethyl acetate	Water	54.7% inhibition of *P. falciparum* at 10 μg/ml	Quinonline alkaloids	[32]
	*Toddalia asiatica* Baill.	Root bark	Methanol	Water	6.8 (chloroquine sensitive, D6); 13.9 (chloroquine resistant, W2)	Furoquinolines (nitidine, 5,6-dihydronitidine), coumarins	[80]
	*Zanthoxylum chalybeum* Engl.	Stem bark	Water	Hot water	4.3 (chloroquine sensitive, NF54); 25.1 (chloroquine resistant, FCR3)	Chelerythine, nitidine, methyl canadine	[28]
Salicaceae	*Trimeria grandifolia* ssp. tropica (Hochst.) Warb.	Leaves	Methanol	Hot water	>50 (chloroquine sensitive, 3D7)		[55]
Sapindaceae	*Blighia unijugata* Baker	Leaves	Ethyl acetate	Hot water	2.3% inhibition of *P. falciparum* at 10 μg/ml		[32]
Simaroubaceae	*Harrisonia abyssinica* Olive.	Roots		Hot water	4.4 (chloroquine sensitive, D6); 10.25 (chloroquine resistant, W2)	Limonoids, steroids	[66]
Solanaceae	*Solanum nigrum L.*	Fruit	Methanol	Hot water	10.3 (chloroquine sensitive, 3D7); 18.7 (chloroquine resistant, K1)	Steroidal alkaloids, flavonoids	[100]
Ulmaceae	*Celtis africana L.*	Stem bark	Ethyl acetate	Hot water	37.5% inhibition of *P. falciparum* at 10 μg/ml		[32]
Verbenaceae	*Lantana camara*	Leaves	Dichloromethane	Hot water	8.7 (chloroquine sensitive, 3D7); 5.7 (chloroquine resistant, W2)	Sesquiterpenes, triterpenes, flavonoids	[30]
	*Lantana trifolia L.*	Arial parts	Petroleum ether	Hot water	13.2 (*P. falciparum*)	Steroids, terpenoids, alkaloids, saponins	[24]
			Ethanol		>50 (*P. falciparum*)		
Zingiberaceae	*Curcuma longa L.*			Hot water; mashing	5 mg/kg had a significantly high chemo suppressive activity of 56.8% (*P. berghei*)	Polyphenolic curcumin	[101]

**Table 4:** Top 17 herbal plants used locally in Uganda for malaria treatment with highest antimalarial/antiplasmodial activities (arranged alphabetically).

Plant family	Plant species	Plant part	Extracting solvent	Report on antiplasmodial, IC_50_ (*μ*g/ml)/antimalarial activity (*Plasmodium* strain)	Active chemical constituents	Toxicity/safety information	Reference(s)
Asteraceae	*Artemisia afra* Jacq. Ex Willd	Leaves	Methanol	3.9 (chloroquine resistant, W2)	Acacetin, genkwanin, 7-methoxyacacetin	Cytotoxicity was observed in Vero cells	[54, 103]
	*Artemisia annua L.*	Leaves	Water	0.9 (chloroquine resistant, W2); 1.1 (chloroquine sensitive, D10)	Sesquiterpenes and sesquiterpene lactones including artemisinin	Generally safe and effective; nausea may occur on drinking herbal extract; artemisinin, an active compound in the extract is safe for pregnant women at least during second and third trimesters	[19, 57, 104]
	*Aspilia africana* (Pers.) C. D. Adams	Leaves	Ethanol	Significant chemo suppressive effect of 92.23% (400 mg/kg) on *P. berghei*	Saponins, terpenoids, alkaloids, resins, tannins, flavonoids, sterols	No signs of toxicity in mice even at a dose as high as 5000 mg/kg	[19, 58]
	*Jatropha curcas L.*	Leaves	Ethyl acetate	2.4 (chloroquine resistant, K1)	Alkaloids, saponnins, glycosides, tannins	Moderate toxicity on thrombocyte line and a protective effect on cardiovascular system; no signs of toxicity in mice following oral administration of 5000 mg/kg body weight (bw) dose	[73, 105]
	*Microglossa pyrifolia* (Lam.)O. Ktze	Leaves	Dichloromethane	1.5 (chloroquine sensitive, 3D7; 2.4 chloroquin resistant, W2)	E-phytol; 6e-geranylgeraniol-19-oic acid	Relatively high cytotoxicity against cells from the human foetal lung fibroblast cell line	[2, 28, 55]
	*Schkuhria pinnata* (lam.)	Whole plant	Methanol	1.3 (chloroquine sensitive, D6)	Schkuhrin I and schkuhrin II	Methanol extract: low cytotoxicity against human cells; aqueous extracts: no observed toxicity observed in mice	[32, 54]
	*Tithonia diversifolia* A. Gray	Leaves	Methanol	1.2 (chloroquine sensitive, 3D7); 1.5 (chloroquine resistant, W2)	Tagitinin C, sesquiterpene lactones	Aerial parts are cytotoxic against cells from the human foetal lung fibroblast cell line	[55]
	*Vernonia amygdalina* delile	Leaves	Methanol/dichloromethane	2.7 (chloroquine resistant, K1)	Coumarin, sesquiterpene lactones including vernolepin, vernolin, vernolide, vernodalin and hydroxyvernodalin, steroid glucosides	Petroleum ether extract shows strong cytotoxicity	[19, 26, 32]
Caricaceae	*Carica papaya L.*	Leaves	Ethyl acetate	2.96 (chloroquine sensitive, D10); 3.98 (chloroquine resistant, DD2)	Alkaloids, saponins, tannins, glycosides	No serious toxicity reported, carpaine, an active compound against *P. falciparum* had high selectivity and was nontoxic to normal RBCs	[65, 106]
Celastraceae	*Maytenus senegalensis*	Roots		1.9 (chloroquine sensitive, D6); 2.4 (chloroquine resistant, W2)	Terpenoids, pentacyclic triterpenes, e.g., pristimerin	No toxicity observed in ethanol extract	[66, 107]
Cucurbitaceae	*Momordica foetida* Schumach.	Shoot	Water	0.35 (chloroquine resistant, FCR3); 6.16 (chloroquine sensitive, NF54)	Saponins, alkaloid, phenolic glycosides including 5,7,4′-Trihydroxyflavanone and kaempferol	No pronounced toxicity against human hepatocellular (HepG2) and human urinary bladder carcinoma (ECV-304, derivative of T-24) cells	[26, 28, 108]
Euphorbiaceae	*Alchornea cordifolia* (Schumach.) Mull. Arg.	Leaves	Water	4.8 (chloroquine resistant, K1)	Phenolics including ellagic acid	No mortality in mice in acute toxicity test	[70, 109]
	*Fluegea virosa* (Roxb. ExWillb.)Voigt	Leaves	Water/methanol	2 (chloroquine resistant, W2)	Bergenin	Nontoxic, extracts exposed to murine macrophages did not slow or inhibit growth of cells	[72, 110]
	*Phyllanthus (pseudo) niruri* Mull. Arg.		Water	Ranged from 2.9 to 4.1 (both chloroquine sensitive, 3D7 and resistant, Dd2)	Coumarins including 1-O-galloyl-6-O-luteoyl-a-D-glucose	No toxicity was observed; thus, LD_50_ of the aqueous extract is >5000 mg/kg. b.w.	[74, 111]
Lamiaceae	*Clerodendrum rotundifolium* Oliv.	Leaves	Methanol	0.02 (chloroquine sensitive, CQ^S^); 1.56 (chloroquine resistant, CQ^R^)	Iridoid glycosides such as serratoside A, serratoside B and monomelittoside, diterpenoids including uncinatone, clerodin, and sugiol	Not explored	[28, 33]
Mimosaceae	*Albizia zygia* (DC.) Macbr.	Stem bark	Methanol	1.0 (chloroquine resistant, K1)	Flavonoids, mainly 3′,4′,7-trihydroxyflavone	The aqueous extract is relatively safe on subacute exposure	[87, 112]
Rubiaceae	*Pentas longiflora* Oliv.	Root	Methanol	0.99 (chloroquine sensitive, D6); 0.93 (chloroquine resistant, W2)	Pyranonaphthoquinones, pentalongin (1) and psychorubrin (2), naphthalene derivative mollugin (3)	Low cytotoxicity	[97]
Rutaceae	*Citrus reticulata*	Seeds (isolimonexic acid methyl ether)		<4.76 (both chloroquine sensitive, D6 and resistant, W2)	Limonin, isolimonexic acid methyl ether, ichangin, deacetylnomilin, obacunone	Dermal 50% lethal dose (LD_50_) of undiluted leaf oil is >2 g/kg in rabbits; seed extract causes respiratory distress and strong spleen contraction	[34, 113]
